# Retaining Doctors in Rural Bangladesh: A Policy Analysis

**DOI:** 10.15171/ijhpm.2018.37

**Published:** 2018-05-05

**Authors:** Taufique Joarder, Lal B. Rawal, Syed Masud Ahmed, Aftab Uddin, Timothy G. Evans

**Affiliations:** ^1^James P Grant School of Public Health, BRAC University, Dhaka, Bangladesh.; ^2^HERD International, Kathmandu, Nepal.; ^3^Centre of Excellence for Health Systems and Universal Health Coverage, James P Grant School of Public Health, BRAC University, Dhaka, Bangladesh.; ^4^International Center for Diarrheal Disease Research, Bangladesh (icddr,b), Dhaka, Bangladesh.; ^5^Health Nutrition and Population, Human Development Network, The World Bank, Washington, DC, USA.

**Keywords:** Health Systems Research, Human Resources for Health, Rural Retention, Policy Analysis, Bangladesh

## Abstract

**Background:** Retaining doctors in rural areas is a challenge in Bangladesh. In this study, we analyzed three rural retention policies: career development programs, compulsory services, and schools outside major cities – in terms of context, contents, actors, and processes.

**Methods:** Series of group discussions between policy-makers and researchers prompted the selection of policy areas, which were analyzed using the policy triangle framework. We conducted document and literature reviews (1971-2013), key informant interviews (KIIs) with relevant policy elites (n=11), and stakeholder analysis/position-mapping.

**Results:** In policy-1, we found, applicants with relevant expertise were not leveraged in recruitment, promotions were often late and contingent on post-graduation. Career tracks were porous and unplanned: people without necessary expertise or experience were deployed to high positions by lateral migration from unrelated career tracks or ministries, as opposed to vertical promotion. Promotions were often politically motivated. In policy-2, females were not ensured to stay with their spouse in rural areas, health bureaucrats working at district and sub-district levels relaxed their monitoring for personal gain or political pressure. Impractical rural posts were allegedly created to graft money from applicants in exchange for recruitment assurance. Compulsory service was often waived for political affiliates. In policy-3, we found an absence of clear policy documents obligating establishment of medical colleges in rural areas. These were established based on political consideration (public sector) or profit motives (private sector).

**Conclusion:** Four cross-cutting themes were identified: lack of proper systems or policies, vested interest or corruption, undue political influence, and imbalanced power and position of some stakeholders. Based on findings, we recommend, in policy-1, applicants with relevant expertise to be recruited; recruitment should be quick, customized, and transparent; career tracks (General Health Service, Medical Teaching, Health Administration) must be clearly defined, distinct, and respected. In policy-2, facilities must be ensured prior to postings, female doctors should be prioritized to stay with the spouse, field bureaucrats should receive non-practising allowance in exchange of strict monitoring, and no political interference in compulsory service is assured. In policy-3, specific policy guidelines should be developed to establish rural medical colleges. Political commitment is a key to rural retention of doctors.

## Background

Despite global, regional and national efforts to improve the health of the general population, the significant challenges remain to meet the health needs of those living in low- and middle-income countries, particularly of those living in rural and hard to reach locations.^1-3^ One of the main underlying factors is the shortage of qualified healthcare providers; also their retention in rural and remote health facilities is problematic.^1,2^ This poses a major challenge for equitable distribution and delivery of health services. Shortages of healthcare providers in rural areas in Bangladesh has a profound impact on access to health services to the large portion of people residing in rural areas.^3,4^

Absolute shortage coupled with internal migration of existing qualified human resources for health (HRH) to urban areas poses an additional challenge against equitable access and use of health services,^1,2,5^ and eventually adoption of universal health coverage.^6^ An adequate number of skilled and motivated healthcare providers is closely associated with improved health outcomes,^7^ which the rural population commonly lack in many countries including Bangladesh.^3^ Retention of doctors in rural areas has been a global problem,^2^ more so for a developing country like Bangladesh. In Bangladesh, there are only 1.1 doctors per 10 000 population in rural areas, compared to 18.2 per 10 000 in urban areas.^8^ The trained healthcare providers, particularly medical doctors, are mainly concentrated in major cities, whereas unqualified or semi-qualified healthcare providers are more skewed to the rural areas, serving the majority of the population in the country.^3,9^ For example, 35% of doctors and 30% of the nurses are serving 15% of the total population living in four major cities of Bangladesh including Dhaka, Chittagong, Rajshahi, and Khulna, whereas less than 20% of health workers serve over 70% people living in rural areas.^3^

Further, there has been a wide gap between sanctioned positions and filled positions.^10^ According to the Bangladesh Health Facility Survey 2014, 62% of the sanctioned positions of doctors are filled at district and sub-district levels; whereas, in more rural parts, such as the union level, the percentage is below 25%.^11^ However, the data available from the Health Bulletin of Directorate General of Health Services (DGHS), 2016 shows that the vacancy rates against sanctioned positions are improving.^12^ Of total 127,841 sanctioned positions of all healthcare providers, 83% positions are filled-up and for doctors, the vacancy rate is 6.88% (out of total 24 028 sanctioned positions). This vacancy rate is likely to be significantly higher in rural health facilities compared to the urban areas.

Considering the needs for addressing rural retention issue, the Government of Bangladesh in recent years has made some significant progress in developing and implementing relevant policies and strategies. Few of them include Bangladesh Health Workforce Strategy 2015,^13^ Human Resource Management (HRM) Operational Plan 2011-2016, Health Nutrition and Population Sector Development Program 2011-2016,^14^ etc. However, these policy provisions are not specifically focused on promoting and retaining health workers, particularly medical doctors in rural health facilities. Further, there are policy provisions to improve rural retention, such as compulsory rural service for newly recruited medical doctors and incentives for rural postings, however, these are not being effectively implemented.

In addition to the policy contents mentioned above, to understand the implementation failures, it is imperative to explore the public policy actors, processes, and contextual issues as well.^15,16^ Although the Bangladeshi health system is governed through different directorate generals (most notably Directorate Generals of Health Services and Family Planning) under the Ministry of Health and Family Welfare (MoHFW), Bangladesh has a pluralistic health system with the coexistence of many stakeholders or agents.^8^ The health sector is served through four major actors: government, private sector, non-governmental organizations (NGOs), and donors. The government sector is responsible for both policy-making (MoHFW) and service provision (directorate generals, such as the DGHS). The government, through DGHS, runs 128 secondary and tertiary level hospitals, 482 upazila and lower level health facilities, and 13 861 community clinics. The private sector is rapidly expanding (currently 78,426 hospital beds in private sector as opposed to 48 934 under DGHS) and is largely unregulated.^12^ This sector constitutes both formal and informal providers, the former targeted towards the rich with high-end services and the latter targeted towards the poor with drugstore based retailing services. NGOs are mostly involved in primary healthcare delivery and donors in technical assistance and financing. Despite pluralistic coexistence of different actors and interest groups, the public health system is highly centralized in the MoHFW, with little delegation (and almost no devolution) of power and responsibilities to the local level.^17^

Recognizing the importance of developing evidence-based policies to improve recruitment, deployment and attraction of skilled health workers to the remote and rural health facilities, World Health Organization (WHO) undertook a major review of the evidence in 2010 and issued 16 policy guidelines on rural retention, grouped under four broad categories of education, regulatory interventions, financial incentives, and personal and professional support.^2^ From among these 16 recommendations, we analyzed three selected rural retention policies. Since the study was conducted in collaboration with the MoHFW, as per the donor’s guideline the priority areas for policy analysis was selected by the MoHFW partners (Human Resource Management Unit of MoHFW; and Center of Medical Education and Health and Manpower Development Unit of DGHS) as: (1) career development programs (recommendation 3.4.4 in WHO guideline, under ‘Personal and professional support’), (2) compulsory services in rural areas (recommendation 3.2.3, under ‘Regulatory interventions’), and (3) schools outside major cities (recommendation 3.1.2, under ‘Education’). This paper aimed at analyzing these policy areas in terms of policy contents, processes, contexts, and the actors.

## Methods

### Research Design

Prior to the selection of key policy aspects, a series of group discussions were held among high officials in MoHFW and researchers from BRAC James P Grant School of Public Health (JPGSPH), and International Center for Diarrheal Disease Research, Bangladesh (icddr,b). Through these discussions, the above-mentioned issues were selected for policy analysis; on the basis that they had been tried in Bangladesh, were relevant to Bangladesh context, and were thought to have a potential impact. For analyzing the policies, we used qualitative methods, which included document reviews; key informant interviews (KIIs) with policy elites, ie, “a specific group of decision makers who have high positions in an organization, and often privileged access to other top members of the same, and other, organizations” (p. 6)^18^; and stakeholder analysis and position-mapping exercise.

### Policy Framework

**Figure 1:**
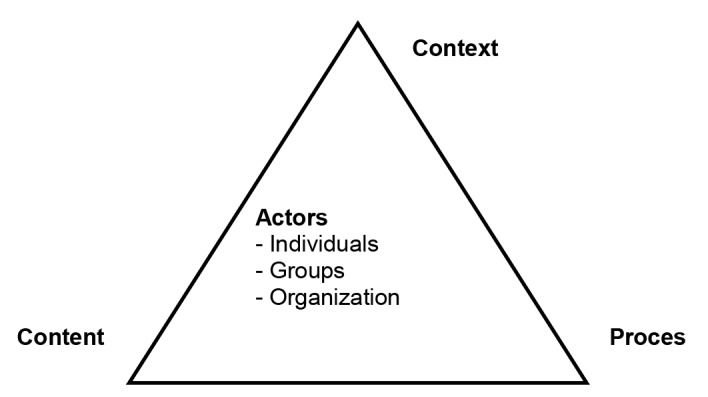
Policy Triangle Framework; source: Walt and Gilson.^15^

Health policy analysis experts suggest that it is a best-practice to base the analysis of policy on an existing framework.^16^ We examined the selected policy areas using the policy triangle framework,^15^ as this is useful for ‘analysis of policy’ (as opposed to ‘analysis for policy’)^18^, ie, understanding an already existing policy. We focused on contest, content, actors, process (Figure 1) relevant to the selected policies:

- Under *Context*, we analyzed, and described the political, social, and cultural factors that might have an influence on the selected policies.
- Under *Content*, we listed the substance of each selected policy.
- Under *Actors*, first we identified the persons or organizations; then assessed their power (authority, financial power, ownership of infrastructure, membership, expertise, legitimacy, access to media, and access to political decision-makers) and position (high opposition, medium opposition, low opposition, neutral, low support, medium support, and high support) in relation to the implementation of specific policies.
- Under *Process*, we discussed the implementation issues related to each policy.

### Search and Review of Relevant Literature

We conducted a search and review of relevant documents, including reports, government documents, conferences and workshop proceedings, and media reports, in addition to journal articles published since 1971 (year of independence of Bangladesh) through May 2013 (year this study was commissioned). The details regarding the search and review process are described in another published article.^19^

### Key Informant Interviews

We conducted KIIs with policy elites working in HRH and related fields in Bangladesh. We identified the potential key informants by series of meetings among the research team members, which included a senior manager (AU) with extensive experience of working in the government health sector as well as research institutions in Bangladesh; and the Dean (TGE) of JPGSPH at the time of the study, with significant experience of working on HRH issues in WHO as well as in Bangladesh. Advice was sought from the researchers of other projects related to HRH issues at JPGSPH.

We employed purposive sampling^20^ strategy, supplemented by snowball method; and stopped data collection after attaining data saturation. Initially we developed a tentative list of nine respondents; but, later, based on the suggestions from them, added two more (one from the DGHS and the other from the Center for Medical Education). Using a semi-structured KII guideline, altogether, 11 KIIs were conducted (Table 1).

**Table 1:** Summary of Key Informants

**Type of Key Informant**	**Number**
High Officials from MoHFW, including DGHS	3
High official of BM&DC	1
Independent consultant of the World Bank	1
Researchers from academic research institutes such as CME, icddr,b, NIPSOM, BIHS, and JPGSPH. One respondent belonged to both icddr,b and JPGSPH.	4
Health journalist of a leading Bangladeshi daily newspaper Prothom Alo	1
High official from administrative cadre of Bangladesh Civil Service	1
Total	11

Abbreviations: DGHS, Directorate General of Health Services; BM&DC, Bangladesh Medical and Dental Council; MoHFW, Ministry of Health and Family Welfare; CME, Center for Medical Education; icddr,b, International Center for Diarrheal Disease Research, Bangladesh; NIPSOM, National Institute of Preventive and Social Medicine; BIHS, Bangladesh University of Health Sciences; JPGSPH, BRAC James P Grant School of Public Health.

The final list of interviewees included policy elites in health sector of Bangladesh, academics, researchers, health journalists, and bureaucrats from both health and outside health sectors. Among the 11 key informants, nine (ie, all but the health journalist and the high official from the administrative cadre) had a long experience of working as a doctor in rural areas of Bangladesh.

The KII guide was drafted by the research team (the first two authors and another person, not included in this manuscript). The draft was then reviewed by more experienced members of the research team (the last two co-authors of this article). Incorporating their feedback, the tool was pre-tested on a colleague, who has experience of working in rural areas of Bangladesh and serving as a health policy and systems researcher at present.

The first author conducted most of the interviews, with exception of one (with a respondent from DGHS), which was conducted by the second author. The interviews lasted from 45 minutes to one hour and were tape-recorded and later translated into English language and were transcribed by one of the study team members. Prior to interviews, the key informants were fully informed about the objectives of the study and the data collection process. Informed written consent was obtained prior to all interviews and for tape recording of the interviews. This study was approved by the Ethical Review Committee of the Bangladesh Medical Research Council.

### Stakeholder Analysis and Position-Mapping Exercise

We conducted stakeholder analysis to systematically gather, analyze, and understand the voice of the policy actors in relation to specific policy areas that we were interested in. Those actors were from MoHFW, NGOs, bilateral agencies, medical colleges, researchers, academia, independent consultants, and health journalists.

Using the steps and the processes recommended by Buse et al,^18^ we also conducted position-mapping exercise among these stakeholders. The position-mapping consisted of the following steps: (1) identifying the policy actors, (2) assessing their political resources or power, and (3) understanding their position and interest with respect to the policy areas. This map included the list of the stakeholders, organized across their positions and color-coded based on their power (Tables 2, 3, and 4). Different interest groups were categorized into different power and positions based on the opinion of the participants of the exercise.

**Table 2:** Position and Power of Stakeholders in Relation to Policies on Career Development Programs

**High Opposition**	**Medium Opposition**	**Low Opposition**	**Neutral**	**Low Support**	**Medium Support**	**High Support**
Bureaucrats from other sectors than health (BCS Administration Cadre, etc.)	Ministry of Finance			Individual doctors	MoHFW	
					DGHS	
					BMA	

Abbreviations: DGHS, Directorate General of Health Services; MoHFW, Ministry of Health and Family Welfare; BMA, Bangladesh Medical Association; BCS, Bangladesh Civil Service.

**Table 3:** Position and Power of Stakeholders in Relation to Policies on Compulsory Services

**High Opposition**	**Medium Opposition**	**Low Opposition**	**Neutral**	**Low Support**	**Medium Support**	**High Support**
	BMA	Individual doctors	Health bureaucrats working at district and sub-district level	Politicians	Media	MoHFW
						DGHS
						Local government

Abbreviations: BMA, Bangladesh Medical Association; DGHS, Directorate General of Health Services; MoHFW, Ministry of Health and Family Welfare; BCS, Bangladesh Civil Service.

**Table 4:** Position and Power Stakeholders in Relation to Policies on Schools Outside Major Cities

**High Opposition**	**Medium Opposition**	**Low Opposition**	**Neutral**	**Low Support**	**Medium Support**	**High Support**
			DGHS		Entrepre neurs (private sector, foundations, NGOs)	MoHFW
						Politicians
						Local government/local people

Abbreviations: DGHS, Directorate General of Health Services; MoHFW, Ministry of Health and Family Welfare; NGOs, non-governmental organizations.

### Data Synthesis and Analyses

We compiled and read all transcripts for data familiarization. To increase the validity, two authors, one with background in health policy and systems research (TJ), and another with background in public health (LBR), independently coded the dataset and reconciled discrepancies through series of meetings. We applied a deductive approach, where *a priori* code-list was developed beforehand, by consulting among the research team. Coded texts were categorized into themes, such as policy content, policy process, policy context, discussion on policy actors, recommendations, and quotations. The themes were then organized across three policy areas such as (*i*) schools outside major cities, (*ii*) compulsory services, and (*iii*) career development program. The information obtained from reviews, KIIs and position-mapping exercise were then analyzed thematically. Manual color coding technique was used for overall data synthesis and analyses. Effort was made to triangulate information from all three data sources.

## Results

### Policy 1: Career Development Programs

#### Contents

**Figure 2:**
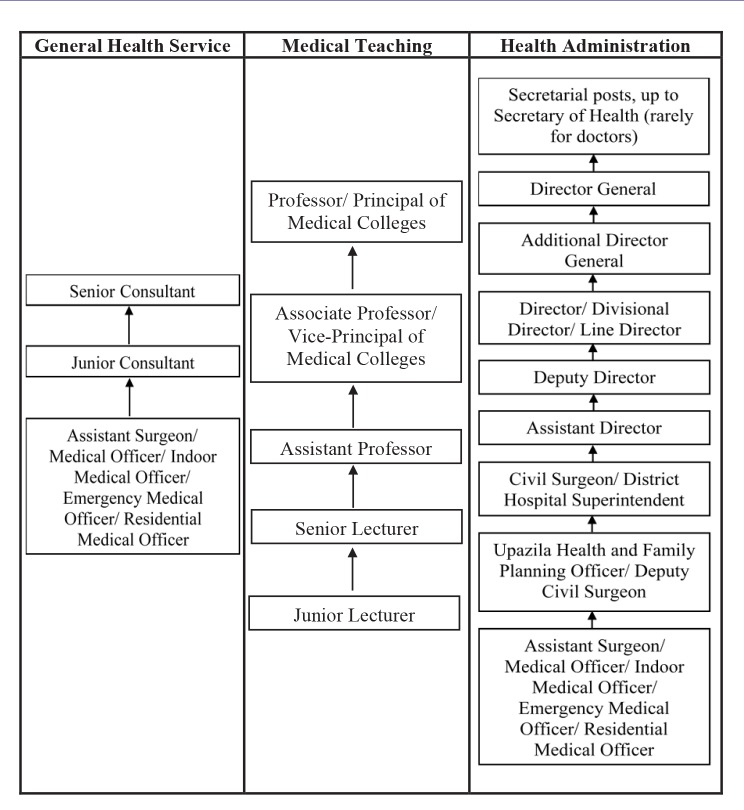
Career Trajectory in Different Tracks Under BCS (Health) Cadre; Adapted from Health Bulletin 2016.^12^

BCS (health) Recruitment Rule, 1981 does not explicitly mention anything about the different tracks under BCS (health) cadre; however, by practice the jobs under this cadre can be classified roughly into three amorphous and often indistinct tracks: (1) General Health Service, (2) Medical Teaching, and (3) Health Administration (Figure 2). Another relevant policy document is the Gazette Notification on Transfer and Posting Policy for Officers in Health Service 2008, which has a provision of providing training and access to higher education; but it does not clearly explain the definitive pathway for career development.

#### Actors

The levels of power of the actors concerning policies for career development program (Table 2) are described below:

MoHFW (high power, medium support): primary policy-making body; generally in favor of career development, but cautious about more competition.

DGHS (high power, medium support): primary implementing body; generally in favor of career development; but traditionally dominated by doctors of Medical Teaching track (mainly from Director up to Director General posts).

Bangladesh Medical Association (BMA) (high power, medium support): political powerhouse of doctors; generally in favor of career development; but more concerned about problems of doctors in Medical Teaching and General Health Service tracks.

Ministry of Finance (MoF) (high power, medium opposition): making any change in the existing career ladder requires MoF’s approval; resistant to any change requiring additional revenue.

Bureaucrats from other ministries (medium power, high opposition): MoHFW posts are often populated by bureaucrats from various other ministries; most of the times without any prior experience or expertise in health sector.

Individual doctors (low power, low support): primary beneficiary of the policy, mainly occupied with clinical practice (mostly aspiring for General Health Service or Medical Teaching tracks); fearful of losing their dominance in higher DGHS positions, should solid career tracks be established. Limited power at policy level.

#### Processes

The MoHFW periodically conducts needs assessment based on the official vacancy of the doctors against the number of available posts. Due to the current insufficient monitoring mechanisms, it is difficult for the MoHFW to determine the actual number of unauthorized absentee doctors; hence the needs assessment for new recruitment often turns out faulty. Based on the requisition from the MoHFW, the Ministry of Public Administration (MoPA) requests the Public Service Commission (PSC) to recruit doctors through Bangladesh Civil Service (BCS) examination. The PSC, which is a quasi-judicial independent commission, recruits doctors along with thousands of other employees for the whole public sector, through BCS examination.

According to PSC job circular, only the doctors with a Bachelor of Medicine and Bachelor of Surgery (MBBS) degree (or equivalent) are recruited as officers in BCS (health) cadre. There is no provision for recruiting or leveraging applicants with additional trainings in allied health disciplines (eg, public health, health systems management, health administration, etc).

According to three key informants and discussants of the position-mapping exercise, after the recruitment, the existing career development policy is not conducive to rural retention as it ensures promotion for only those who has a post-graduation. That promotion also comes relatively late in Health Administration compared to Medical Teaching and General Health Service tracks. This gridlock can only be broken by doing a post-graduation, which again requires the doctors to stay in urban settings. The post-graduation training also paves the way for pursuing the career in either Medical Teaching or General Health Service tracks, leaving the Health Administration track ignored and deprived. A key informant from DGHS said,

“*If doctors stay in rural areas for long time, they forget everything they learned in medical schools. The need for post-graduation encourages the doctors to stay in major cities or at least the district level.”*

A fundamental principle of career development programs is that anybody entering a cadre service must have the scope to climb up to the highest level. But this principle is not adhered to in health service cadre. According to the respondent from MoHFW, doctors, after being recruited through BCS examination of PSC, undergo foundation training with other civil servants. Only after serving the government at the entry level post (known as Medical Officers or Assistant Surgeons) for 4/5 years and obtaining a higher degree, doctors can choose one of the three tracks. The career tracks being intensely porous, the migration from one track to another at various stages of career is also common. According to the respondent from a research organization, who also has a long career as doctor under DGHS, track changes are most common between Medical Education and General Health Services. Highest posts of DGHS are traditionally filled up by doctors by lateral migration from either Medical Education or General Health Service tracks; usually not by vertical promotion within Health Administration track itself.

The supreme policy-making body in Bangladesh health sector is the MoHFW, where doctors from any of the health cadre tracks are very rarely deployed, as stated by seven respondents from DGHS (2), BMDC, independent consultant, and the academic institutions (3). According to them, running the ministry requires solid administrative skills, management knowledge, leadership capabilities; which arguably cannot be managed on makeshift basis by untrained professionals. Due to the existing recruitment policy described at the beginning of this section, MoHFW reportedly never finds adequate staffs with required qualifications to run the ministry. The Secretary of Health, who is the supreme administrator of MoHFW, is almost always borrowed from other BCS cadre services than the Health. This person who comes to this vital position usually never has experience of working in health sector, nor does he have any formal training in public health or health systems management. When asked about the effectiveness of placing an administrator from a different ministry as the head of MoHFW, and whether this is a right thing to do, the key informant from MoPA said:

“*Administrators under BCS Administration Cadre are trained to take on any kind of responsibilities. It is all about getting people to do their jobs, which does not require much technical know-how’s about that particular field. MoPA is often headed by a person formally trained in engineering; Ministry of Foreign Affairs has a good number of doctors. Then what is the problem if MoHFW is headed by someone from other ministries? MoHFW is nothing special!”*

On top of career tracks being porous and poorly planned, career development or promotion of doctors are often influenced by political interferences. The manipulation of promotion of the civil servants, and even politicization the PSC itself has been criticized and reported both in the media and the academic publications.^21-23^ The promotion rules have been altered numerous times in favor of political manipulations. In recent past, Bangladesh Civil Service, specially the health sector, experienced the bypassing of the promotion rules. This caused the doctor political leaders of the ruling party to gain undue promotions, particularly in the Medical Teaching track. The BMA leaders of ruling party, by virtue of their political affiliation, succeeded in gaining promotion for a large number of their supporters, who were allegedly ineligible for the post. When these news came to the media,^21^ it invoked criticisms and even protests from the deprived doctors; and the whole issue ended up in a litigation in the high court.

### Policy 2: Compulsory Services

#### Contents

The compulsory service of doctors in rural health facilities has been implemented since 1980s. In 2008, the government issued a revised gazette notification known as ‘Transfer and Posting Policy for Officers in Health Service.’^24^ According to this policy, the newly appointed doctors must serve at least two years in rural areas. There are no special provisions for newly deployed female doctors, however, in case of a couple both of whom are doctors, consideration (not priority!) is given for posting them at the same station.

#### Actors

The levels of power of the actors concerning policies for compulsory services (Table 3) are described below:

- MoHFW (high power, high support): formally highest policy-making body, interested to materialize government’s mandates and explicit policies.
- DGHS (high power, high support): deploying doctors and ensuring their services are primary responsibility.
- BMA (high power, medium opposition): enjoys membership of almost all Bangladeshi doctors, has high political stakes and involvements at state level. BMA has shown their power in various occasions in the forms of strikes, refraining from providing medical care in the hospitals, demonstrations, and even physical confrontations. Their power is often claimed to be so high as to shake the government itself.^25,26^
- Media (medium power, medium support): Media plays a very important and vibrant role in reporting absenteeism.
- Politicians (medium power, low support): demonstrates contrasting roles—when approached by local people, they stay in favor of compulsory service. Contrarily, when approached by the political body of the doctors, they keep their mouth shut on this issue.
- Health bureaucrats working at district and sub-district level (medium power, neutral support): responsible for monitoring doctors.
- Local government (low power, high support): vocal supporter of this policy, but has hardly any power to exercise.
- Individual doctors (low power, low opposition): naturally are not interested to carry out compulsory rural service; but, despite being the primary stakeholder of this policy, individually they have little power to exercise.

#### Processes

The article 25 of the Government Servants (Conduct) Rules, 1979 maintains, *“No Government servant shall be a member of, or be otherwise associated with, any political party or any organization affiliated to any political party, or shall take part, or assist, in any manner, in any political activity in Bangladesh or abroad.”*^22,27,28^ However, many high officials of DGHS are affiliated (if not directly involved) with BMA politics. It is alleged that, doctors affiliated with the ruling government faction of BMA can bypass the compulsory service. Since the government puts doctors in the administrative hierarchy often based on their political affiliation, these officials are also reluctant to go against the interest of BMA to obligate the doctor of the same faction to serve compulsorily in rural areas. One key informant said:

“*Doctors are sent to rural areas by one government order; then again withdrawn from those areas by another government order. This is a mystery how these government orders are made.”*

The health bureaucrats working at district and sub-district level often relax their monitoring in implementing the compulsory services, either due to political alignment or vested interests.^29^ They find themselves in difficult position regulating the rules of compulsory deployment because of political pressures.

One thing that may potentially change this situation is to improve the monitoring mechanism, which was attempted by the Management Information System (MIS) division of DGHS. They introduced biometric fingerprint attendance machines on pilot basis and was planning to scale it up to all sub-districts. This initiative, despite being commended by various civilian groups, allegedly, started facing challenges immediately after implementation. During the pilot phase, BMA election came up, and it is alleged by one of our key informants that BMA leaders of the ruling party asked the relevant bureaucrats not to make too much of it, fearing losing of votes.

Apart from political pressures, health bureaucrats working at district and sub-district level themselves often turn a blind eye on the absentee doctors, as absenteeism is deemed mutually beneficial. With their tacit approval, instead of constantly staying in rural posts, doctors go to rural health facilities only on roster dates. This arrangement leaves many local patients for those bureaucrats, who are engaged in private practice in addition to public job.

Many of the newly recruited doctors, who the compulsory service policy is targeted for, are posted at Union Sub-centers (health facilities even below the sub-district level), which lack even the basic amenities, infrastructure, housing, security and transportation, as reported by the five respondents (the health journalist, one DGHS respondent, independent consultant, two respondents from the academia). The DGHS respondent said,

“*There are some limitations in our part. We do not have sufficient accommodation to keep them (newly recruited doctors) in the rural center.”*

While asked why the government might have created the posts without facilities, the independent consultant key informant reported that, people at different levels of bureaucracy, in tandem with some politicians, receive money from the applicant doctors, in exchange of an assurance to get them a job. He added the comment,

“*Every recruitment is a purchase, and every purchase has a commission.”*

#### Policy 3: Schools Outside Major Cities

##### Contents

There are no specific policy documents to obligate either the public or private sector to establish medical colleges outside the major cities. The Rules for Private Medical and Dental Colleges in Bangladesh, 2013 suggests the private sector to expand their education services throughout the country, without mentioning specifically to establish schools outside major cities.

##### Actors

The levels of power of the actors concerning schools outside major cities (Table 4) are described below:

- MoHFW (high power, high support): decides formally where a public medical college will be established.
- Politicians (high power, high support): in absence of specific policy document, politicians use political power to establish medical college in own constituency.
- Entrepreneurs (medium power, medium support): individual entrepreneur, the philanthropic foundations, or NGOs decide where to establish a private medical college.
- DGHS (medium power, neutral support): responsible for arrangement of intake exams, deployment of teachers; overall implementation of decisions.
- Local government/ local people (low power, high support): has interest and high demand, but has no formal way of exercising the power.

##### Processes

Although according to the guideline for establishing medical colleges there should be at least an accompanying 250-bedded hospital (or five beds per medical student), the decision often does not depend on existence of a suitable hospital, rather on political decision. Participants of the position-mapping exercise as well as most of the key informants supported this statement. The key informant from DGHS said,

“*I am telling you very frankly that this (decision to establish a public medical college) is totally political. The political leader who have power and good command with high profile raise the demand to establish a medical college with or without any justification.”*

One of the key informants informed that the medical colleges were established in the constituencies of the influential Members of Parliament of the ruling parties. One respondent (the independent consultant) categorically mentioned,

“*That’s why you see a medical college in District X [ie, the District the Head of Government at the time of the study was hailing from].”*

This, however, does not hold for private medical colleges. It is the entrepreneurs’ choice where they want to establish private medical colleges; and they do it where profits can be maximized.

A summary of the policy analysis is provided in Table 5.

**Table 5:** Summary of Policy Analysis

	**Contents**	**Actors**	**Processes**	**Policy Recommendations**
**Policy 1: Career development program**	BCS (health) Recruitment Rule, 1981 Indistinct tracks: 1. General Health Service, 2. Medical Teaching, 3. Health Administration Gazette Notification on Transfer and Posting Policy for Officers in Health Service 2008	MoHFW (high power, medium support) DGHS (high power, medium support) BMA (high power, medium support) Ministry of Finance (high power, medium opposition) Bureaucrats from other departments (medium power, high opposition) Individual doctors (low power, low support)	Porous tracks Not leveraging applicants with required skills Late promotions Promotion contingent on post-graduation in clinical field only People from other ministries occupying high MoHFW posts Higher DGHS posts filled up by lateral migration from other tracks Change of promotion system to facilitate political interference Undue promotion to political allies	Applicants with expertise and experience in public health, health systems, health administration, etc should be given leverage, or recognition in BCS (health) recruitment Recruitment should be quick, customized, and transparent, without scope for graft and political influence or motive Tracks within the health service (General Health Service, Medical Teaching, and Health Administration) must be clearly defined, distinct, and respected New recruits should be assigned to one track from the beginning Lateral entry from other tracks should be restricted Promotion within each track should be timely, smooth, fair, and free from corruption and political interference Higher education should be encouraged and rewarded, but not at the cost of rural postings
**Policy 2: Compulsory services**	Compulsory service in rural areas for 2 years, in law since 1980s Transfer and Posting Policy for Officers in Health Service, 2008 Not conducive for couple doctors	MoHFW (high power, high support) DGHS (high power, high support) BMA (high power, medium opposition) Media (medium power, medium support) Politicians (medium power, low support) Health bureaucrats working at district and sub-district level (medium power, neutral support) Local government (low power, high support) Individual doctors (low power, low opposition)	No priority for females to be posted in the same location as their spouse Health bureaucrats working at district and sub-district level allowing doctors’ roster duty Creating posts without facilities to take money against recruitments Bending the rule due to political affiliation	Amenities, equipment, infrastructure, security, and other facilities should be ensured prior to posting These should accommodate current trend of feminization of medical profession Female employees should be prioritized to stay with their spouse Strict monitoring of staff, using modern technology Scrutinize field bureaucrats Non-practising allowance for field bureaucrats Bypassing of compulsory services based on political alliance must be stopped Separation of politics from bureaucracy must be encouraged Existing rule prohibiting public servants from getting engaged in politics must be enforced
**Policy 3: Schools outside major cities**	No specific policy Rules for Private Medical and Dental Colleges in Bangladesh, 2013: private sector to expand education throughout country	MoHFW (high power, high support) Politicians (high power, high support) Entrepreneurs (medium power, medium support) DGHS (medium power, neutral support) Local government/local people (low power, high support)	Absence of a policy to establish schools outside cities Private entrepreneurs establishing medical schools on profitability, not rural retention considerations Establishing medical colleges using political influence	Specific policy guideline should be prepared This should have specific directions for both public and private sector Population need, rather than profit or political motive must get priority in deciding the site Rural students should get priority in admission

Abbreviations: DGHS, Directorate General of Health Services; MoHFW, Ministry of Health and Family Welfare; BMA, Bangladesh Medical Association; BCS, Bangladesh Civil Service.

## Discussion

The analysis of three policies related to rural retention of doctors revealed four cross-cutting themes: lack of proper systems or policies in some areas, vested interest or corruption of stakeholders aggravating the situation, undue and all pervasive political influence, and configuration and power of those in places of importance for policy-making. These are discussed below with implications for the ‘rural retention’ problem in Bangladesh.

### Absence of Proper System or Policy

In policy area 1, ie, career development programs, we found, despite the existence of policy provision for career path development, principles for promotion and transfer, and provision of post-graduation and in-service training to the medical doctors, these policy provisions are neither well defined nor well implemented when it comes to career development of doctors, particularly for those working in the rural areas. This has also been observed in earlier studies from Bangladesh.^25,30^ No leverage was given to applicants with relevant qualifications (eg, public health, health systems management, health administration) during recruitment in BCS (health). Promotions were late in Health Administration track and largely contingent on clinical post-graduation, which reduced the duration of rural stay. These findings are also supported by Darkwa et al.^30^

In policy area 2, ie, compulsory services, we found females were not given the priority to work in the same location as their male doctor partners. In Bangladesh context, many of the female doctors’ partners were from the same profession and residing in rural areas without their male partners was often socially unacceptable.^9,31^ Similar pattern was observed in Pakistan, which shares fair social, cultural, religious, and historical similarity with Bangladesh.^32,33^

In policy area 3, ie, school outside major cities, we found there was no explicit policy document to encourage or enforce establishment of medical colleges in rural areas. Absence of proper health systems support and policy promoting rural retention has also been reported in several other countries.^9,30,32^

### Vested Interest or Corruption

In policy area 1, we found personnel from other ministries get lateral entry in occupying high MoHFW posts. DGHS posts were also filled up by doctors from tracks other than the Health Administration track. This tendency is arguably neither new nor special to Bangladesh.^25,34^

In policy area 2, we found field level health bureaucrats facilitated doctors’ departure from duty station (by allowing roster based non-continuous duty) in the interest of their own private practice. This type of tolerance towards partial or total absenteeism in public sector is observed in some African countries as well.^35^ The findings also show that some higher-level bureaucrats and politicians created posts for doctors without accounting for required infrastructure and facilities; allegedly to graft money from applicants in exchange of ensuring those jobs. The importance of proper infrastructure and facilities in favor of rural retention has been widely reported in literature,^9^ and the issue of bribery in exchange of job offers in health sector has been widely reported in Bangladeshi media.^36^

In policy area 3, we found private entrepreneurs established medical colleges based on financial motives, rather than consideration for rural retention. The profit motive of private higher educational institutions is well documented,^37,38^ and understandable.

### Political Interference

In policy area 1, we found recent changes in promotion system to facilitate political interference. We also found evidence of undue promotions motivated by political affiliations, leading even to litigation. Shah et al also showed, in Pakistan, politically powerful persons frequently interfered with appointment and transfer of health sector staff.^32^ Similar pattern was observed in Sierra Leone, where job postings are reportedly altered due to political interference and deserving candidates are often deprived of promotions due to political motives.^39^

In policy area 2, the participants shared that members or the activists of ruling party faction of BMA generally bypass the provision of ‘compulsory’ rural services. Darkwa et al also showed, in Bangladesh, absentee doctors maintained good relations with higher authorities and politicians, and obtained unfair recommendations from them to avoid compulsory rural services.^30^ Similar ‘political trade-unionism’ was observed before, when policy attempts were made to restrict doctors’ private practice and introduce local government-led monitoring mechanism of the health centers.^25^

In policy area 3, we found politics or the politicians, not the policy guidelines, determined where a medical college would be established. Overall, political factors have been found to be demotivating for doctors to stay in rural areas.^9,32^ Political scientists Jahan and Shahan, drawing examples from different sectors of Bangladesh, argued that the power of politics is something we cannot just ignore.^23^

### Position and Power of Actors of Interest

In policy area 1, we found high opposition of bureaucrats against career development of doctors. This was understandable, because, proper career development within MoHFW (ie, from field level up to the top) would mean their opportunities of posting at the top positions in a ministry would be compromised. Individual doctors were not much supportive too, as rural posting meant decreased quality of life and career opportunities.

In policy area 2, the findings suggested that, in most cases the BMA showed medium opposition to compulsory services, as many of their members would not want to go to rural areas. Dussault and Franceschini also showed how strategies to deal with geographical distribution of HRH faced negative outcomes due to resistance from professional groups of doctors in other countries.^9^ Health bureaucrats working at district and sub-district level remained neutral, torn between their professional responsibility and political alignment. Interestingly, local politicians also showed low support, as opposed to the expectation that they would be highly supportive due to the demand of the people of their constituency. Local government had a high support for the policy, but they held low power in implementing the policy.

In policy area 3, we found high support and power of MoHFW and politicians for establishing schools outside major cities, which was encouraging. But private entrepreneurs had medium support, as their support often depended on profit motive. Local government/local people enjoyed low power in this regard, despite their high support for the policy.

### Policy Recommendations

Roberts, Hsiao, Berman, and Reich suggest that position, power, players, and perceptions of the policy stakeholders have important bearing on policy implementation.^40^ Strategies, if designed based on the understanding of the position and power of actors of interest, can impart a positive influence on the rural retention of doctors.^18^ Considering these factors, and drawing on our findings from the policy analysis, we propose the following policy recommendations:

In policy area 1, to ensure a smooth career development, first, the recruitment policy needs to be updated considering the changes and the current needs of the Bangladesh health systems. Doctors with expertise and experience in public health, health systems, health administration, etc. should be given leverage, or at least recognition. Recruitment should be quick, customized, and transparent, without scope for graft and political influence or motive. Secondly, the tracks within the health service (General Health Service, Medical Teaching, and Health Administration) must be clearly defined, distinct, and maintained. New recruits should be assigned from the very beginning to one of these tracks, with possibility of track changes only on special circumstances. Lateral entry from other tracks, especially in high ranks should be restricted. This principle should apply to MoHFW positions, up to the highest level. Thirdly, promotion within each track should be timely, fair, and free from corruption and political interference. It has been found that measures such as organizational support like timely promotion has a positive association with staff satisfaction (Pearson correlation coefficient 0.37) while it has a negative association (-0.42) with turnover intention of Bangladeshi public-sector doctors,^41^ eventually impacting on rural retention. Finally, higher education should be encouraged and rewarded, but not at the cost of rural postings.

In policy area 2, to ensure compulsory services, first, the security, amenities, equipment, infrastructure, and other facilities should be ensured prior to posting. These should be in keeping with the current trend of feminization of medical profession in Bangladesh. Accounting for the socio-cultural reality of Bangladesh, female employees should be prioritized to stay with their spouse, if applicable. Secondly, once posted, they should be strictly monitored; modern technology (eg, biometric finger print, online sign-in and random central monitoring, etc) may be employed in this regard along with bringing under strict control of the health bureaucrats working at district and sub-district level with clearly defined roles and responsibilities for them. Non-practising allowance may be considered to engage them for better management. Thirdly, bypassing of compulsory services based on political alliance must be stopped. For this to implement, separation of politics from bureaucracy is encouraged. Existing rules prohibiting public servants from getting engaged in politics must be enforced.

In policy area 3, to establish medical schools outside major cities, first, specific policy guidelines on establishing medical colleges should be prepared with clear directions for both public and private sectors. Actual population need, rather than profit motive or political ‘sweet will,’ must get priority for establishing medical colleges. The rural students in real term should get priority in getting admission in these institutions.

### Strengths and Limitations

The research was greatly benefitted by the team combination of both ‘insiders’ (from Bangladesh - TJ, AU, SMA) and ‘outsiders’ (not from Bangladesh - LBR, TGE), a combination that reportedly yields “the richest and most comprehensive understanding of the policy process.”^16^ However, the focus of the study was limited to the doctors working in the formal health sector of Bangladesh, and also higher number of respondents from a more diverse background including those working at the district/sub-district level could not be interviewed due to time and resource constraints. Private-sector doctors, despite their increasing dominance in health service provision, were not included in this analysis. This was because we tried to focus the policy analysis in alignment with the priority concern of the MoHFW, which was ‘absenteeism’ of public-sector doctors in public health facilities in the rural areas.

## Conclusion

The crisis of retaining doctors at their places of rural postings often stemmed from weaknesses of the health systems and its policy environment. Often the effectiveness of existing policies was compromised by failures in implementation due to political interference and corruption. Our position-mapping exercise revealed that, some policy makers in high positions (eg, bureaucrats from other ministries than health, BMA) opposed some of the rural retention policies, whereas those who supported (eg, local people, local government) were not sufficiently empowered. Ultimately, commitment from the highest level of political hierarchy is the key to the successful implementation of the rural retention policies of the government.

## Acknowledgements

This study was conducted in collaboration with the Human Resource Management Unit of the MoHFW, DGHS, and BRAC James P Grant School of Public Health (JPGSPH), BRAC University, Dhaka, Bangladesh. The study was technically supported and funded by WHO Bangladesh under the BAN HRH program. We thank research team members Dr. Kawkab Mahmud, Dr. MD. Islam Bulbul (from DGHS), Mrs. Iffat Nowrin Tuly, and MD. Tarek Hossain for their contribution for project coordination, data collection, and translation. We also thank Prof. Dr. ABM Abdul Hannan (from DGHS) for his overall supervision while conducting this study. Last but not the least, thanks go to those policy makers and key informants who provided their valuable time and information to this study.

## Ethical issues

The research was reviewed and approved by the National Research Ethics Committee of the Bangladesh Medical Research Council.

## Competing interests

Authors declare that they have no competing interests.

## Authors’ contributions

TJ designed the methodology, developed the tools, collected data, analyzed data, and wrote the manuscript. LBR contributed in developing the methodology, tools, data collection and analysis, manuscript review and editing. SMA reviewed manuscript and provided inputs. AU suggested key informants and policy documents, and also reviewed the manuscript. TGE contributed in developing the methodology and provided overall conceptual support.

## Authors’ affiliations

^1^James P Grant School of Public Health, BRAC University, Dhaka, Bangladesh. ^2^HERD International, Kathmandu, Nepal. ^3^Centre of Excellence for Health Systems and Universal Health Coverage, James P Grant School of Public Health, BRAC University, Dhaka, Bangladesh. ^4^International Center for Diarrheal Disease Research, Bangladesh (icddr,b), Dhaka, Bangladesh. ^5^Health Nutrition and Population, Human Development Network, The World Bank, Washington, DC, USA.

## Key messages

**Implications for policy makers**

- The recruitment policy of the health cadre of Bangladesh Civil Service should be changed in such a way that leverages applicants with training in public health, health systems, health management, etc., in addition to the existing qualifications.
- Recruitment and promotion of doctors should be timely, customized (ie, according to the specific need of the health cadre of Bangladesh Civil Service), and transparent.
- Career tracks (General Health Service, Medical Teaching, Health Administration) are found to be porous and unplanned; so, these need to be clearly defined, distinct from one another, and respected (ie, free from political motives or motives other than relevant expertise and experience).
- Health bureaucrats working at district and sub-district level reportedly relax their monitoring for personal gain or political pressure; so, they should receive non-practising allowance, so that they are encouraged for stricter and better monitoring.
- Since medical colleges are often established based on political consideration (public sector) or only profit motives (private sector), specific people-centered policy guidelines should be developed and implemented regarding establishing rural based medical colleges.

**Implications for the public**

This research unearths several loopholes contributing to the failures in retaining doctors in rural areas. These findings would empower the citizen to enhance their vigilance on the public-sector doctors and their managers, and hold them accountable for the services they are mandated to provide.
